# A highly sensitive and green electroanalytical method for the determination of favipiravir in pharmaceutical and biological fluids

**DOI:** 10.1186/s13065-023-01023-z

**Published:** 2023-08-31

**Authors:** Heba M. El-Sayed, Hisham Ezzat Abdellatef, Hassan A. M. Hendawy, Omar M El-Abassy, Hany Ibrahim

**Affiliations:** 1 Analytical Chemistry Department, Faculty of Pharmacy, Zagazig University, Zagazig, 44519 Egypt; 2 Egyptian Drug Authority, Giza, Egypt; 3 Pharmaceutical Chemistry Department, Faculty of Pharmacy, Egyptian Russian University, Badr, 11829 Egypt

**Keywords:** Favipiravir, Reduced graphene oxide, Square wave voltammetry, Cyclic voltammetry

## Abstract

**Background:**

Favipiravir is currently used for the treatment of coronavirus disease-2019 (COVID-19).

**Objective:**

A highly sensitive and eco-friendly electroanalytical method was developed to quantify favipiravir.

**Method:**

The voltammetric method optimized a sensor composed of reduced graphene oxide / modified carbon paste electrode in the presence of an anionic surfactant, improving the favipiravir detection limit. The investigation reveals that favipiravir-oxidation is a diffusion-controlled irreversible process. The effects of various pH and scan rates on oxidation anodic peak current were investigated.

**Results:**

The developed method offers a wide linear dynamic range of 1.5–420 ng/mL alongside a higher sensitivity with a limit of detection in the nanogram range (0.44 ng/mL) and a limit of quantification in the low nanogram range (1.34 ng/mL).

**Conclusion:**

The proposed method was applied for the determination of favipiravir in the dosage form, human plasma and urine samples. The developed method exhibited good selectivity in the presence of two potential electroactive biological interferants, uric acid which increases during favipiravir therapy and the recommended co-administered vitamin C. The organic solvent-free method greenness was evaluated via the Green Analytical Procedure Index, The present work offers a simple, sensitive and environment-friendly method fulfilling green chemistry concepts.

**Supplementary Information:**

The online version contains supplementary material available at 10.1186/s13065-023-01023-z.

## Introduction

**Fig. 1:**
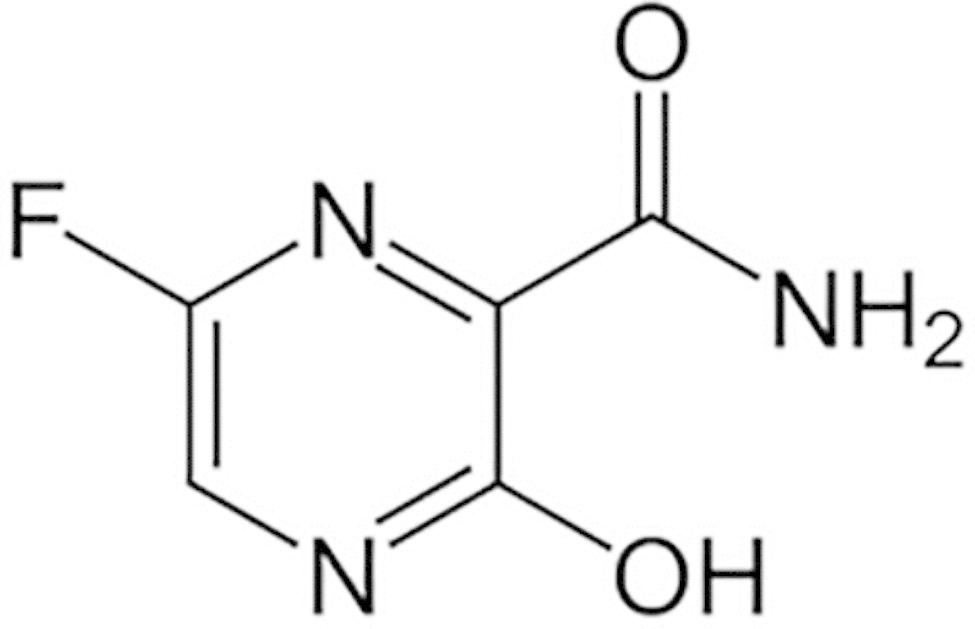
Chemical structure of FAV

The coronavirus epidemic (COVID-19) was first announced in late December 2019. Since then, it has rapidly spread all over the globe creating a huge pressure on public health systems. According to a report from the World Health Organization (WHO), there were 203,295,170 confirmed cases and 4,303,515 confirmed deaths worldwide as of August 10th, 2021. The countries with the highest excess mortality rates were the United States (640,000 by June 6, 2021), Russia (500,000 by April 30, 2021), Brazil (500,000 by May 31, 2021) and Mexico (470,000 by May 23, 2021) [1]. As a quick response, a number of already approved and marketed drugs, including Favipiravir (FAV), have been tested and implemented in the treatment of the coronavirus disease [2]. FAV, a purine nucleic acid analog (Fig. 1), is chemically known as 6-fluoro-3-hydroxy-2-pyrazinecarboxamide. FAV inhibits effectively and selectively the ribonucleic acid (RNA)-dependent RNA polymerase of RNA viruses [3]. FAV displayed a good activity against a wide range of different influenza viruses. Moreover, it inhibits influenza strains resistant to current antiviral drugs [4, 5]. It is an oral medication that was approved in 2014 for new and re-emerging influenza pandemics [6, 7]. Recent evidences demonstrated that FAV has an inhibitory effect against COVID-19 [8]; and subsequently, it was recommended as an emergent medication in several countries [9].

Several analytical methods were reported for the determination of FAV including chromatographic [10–15] ,spectrofluorimetric [14, 16] and voltammetric methods [17–22]. However, voltammetric methods, compared with high performance liquid chromatography (HPLC) and spectrofluorometric methods, have the advantages of being sensitive, rapid and do not require hazard organic solvents or derivatization; such characteristics are important from practical and economical point of views. Square wave voltammetry (SWV) is one of the fastest and most sensitive pulse voltammetry techniques. Square wave and differential pulse voltammetry, for instance, have been employed over past decades for the rapid determination of several pharmaceutical ingredients [23–28]. Moreover, electrochemical sensors with enhanced sensitivity have been achieved benefiting from the new physicochemical properties of nanomaterial’s, such as high surface area, electrical conductivity, excellent chemical stability and outstanding electrocatalytic properties. Some electrochemical methods were recently reported for the analysis of FAV. An electrochemical sensor based on a molecularly imprinted polymer was reported for the analysis of FAV in biological fluids, but the method requires sample pretreatment and drying under nitrogen [18]. Other methods suffer from expensive materials as diamond or gold, and/or lengthy electrochemical sensor preparation [19, 20, 22]. Recently, Mohamed et al. [21] proposed manganese oxide-reduced graphene oxide-based electrochemical sensor for detecting FAV in dosage forms and plasma samples. Despite its remarkable selectivity, the combination of manganese dioxide and reduced graphene oxide did not provide a much lower limit of detection than proposed study. The most recent electroanalytical report displayed a relative lower sensitivity within the micromole range [17]. To this end, electrochemical sensors modified with graphene or reduced graphene oxide (RGO) have been used for the identification and determination of several organic, inorganic, and biological important molecules [29].

The environmental impact is a crucial factor that has to be considered when developing a new analytical procedure for a particular analyte. Thus, a comprehensive assessment of the greenness of all solvent and chemical involved in the experimental work is required and should be a main part of the developed method. Nowadays, Green Analytical Procedure Index (GAPI) [30] metric is considered to be sufficient to discuss the extent of chemical safety and the weaknesses of any applied analytical method in order to minimize any harmful dominance to the personnel and the environment, and to aid in further improvement or modification.

Herein, we report a sensitive and rapid voltammetric procedure for the determination of FAV. This method depends on the electrochemical oxidation of FAV using SWV at a reduced graphene oxide electrode. Several experimental parameters that are crucial for the electrochemical sensitivity to FAV were optimized. The method was applied to determination of FAV in human plasma and urine without fear of interference. Vitamin C (Vit. C) is an important water-soluble vitamin, antioxidant and free radical scavenger. It was reported that vitamin C in serum and leukocyte levels were depleted during acute stage of the COVID 19 infection [31, 32]. Vit. C was recommended and introduced as an adjunctive and a supportive treatment for COVID 19 [33]. Therefore, possible interference of co-existing electroactive compounds such as Vit C with FAV determination was investigated in the present work. Another potential electroactive interferant is the uric acid whose blood level was reported to be increased as a frequent side effect of FAV [34].

The benefits of our unique technique prompted our research team to develop voltametric procedures for evaluating FAV in dosage form and real samples. In addition, a rapid and sensitive approach for trace FAV detection is still desired, and despite these and other attempts in the literature [17–22], efficient, alternative methods are still required to suit the needs of a broad variety of sensing applications. Also the greenness of the method was assessed using GAPI metric.

## Experimental

### Instrumentation

The voltammetric experiments were performed using 797VA Computrace Metrohm potentiostat which is provided with 797VA Computrace software version 1.3. The electrochemical cell consists of three electrodes: the working electrode (laboratory-made (RGO/carbon paste electrode), the reference electrode is an Ag/AgCl (3 M KCl) and the counter electrode is a platinum wire. All pH measurements were carried out using a JENWAY 3510 pH meter. Minitab software was used for comparison. The measurements were carried out at an ambient temperature (25 ± 0.1 °C). A sonicator (Model WUC-A06H, manufactured by DAIHAN Scientific Co. Ltd.) was used in this experiment.

### Chemicals and reagents

All reagents were analytical-reagent grade and used without further purification. Deionized water was used as a diluent throughout the present work unless otherwise stated.

FAV standard (M.wt: 157.1, purity: 99.5%) was a kind gift Egyptian Drug Authority. Avipiravir® 200 mg tablets, Eva Group Limited, Cairo, Egypt, was obtained from local market. Vitamin C, Uric acid and the dosage form excipients as mannitol, magnesium stearate and carboxy methylcellulose sodium were obtained from the Egyptian Drug Authority (EDA). Stock Britton Robinson (BR) buffer solution 0.04 M was prepared according to a published procedure [35]. Graphite powder (particle dimension > 20 µM), reduced graphene oxide, sodium dodecyl sulphate (SDS), potassium ferrocyanide trihydrate K_3_Fe(CN)_6_.3H_2_O, paraffin oil, boric acid, sodium hydroxide (NaOH), orthophosphoric acid, and glacial acetic acid have been purchased from Sigma-Aldrich. BR buffers with different pH (2–10) were prepared from the stock buffer solution by adjusting the pH using 0.2 M aqueous NaOH. Human plasma was provided by Zagazig University Hospitals, Zagazig, Egypt, and was stored frozen until used following a moderate thawing. Urine samples were taken from healthy male volunteers and kept frozen until needed.

### Standard and working solutions

A primary stock standard solution of FAV was prepared at a concentration of 100 µg/mL via dissolving accurately 10.0 mg of FAV in 100-mL volumetric flask and completing the volume with 0.01 M aqueous NaOH. The secondary stock standard solution (500 ng/mL) was prepared from its primary solution by transferring 0.5 mL into another 100-mL volumetric flask and completing the volume with the same solvent (0.01 M aqueous. NaOH). The working standard solutions (1.5–420 ng/mL) were prepared from the secondary standard solution using the same solvent and subjected to voltammetric measurements.

### Working electrodes

The carbon paste electrode (CPE) was prepared by mixing 250.0 mg graphite powder with 90 µL of paraffin oil using a mortar and a pestle. A portion of the carbon composite was packed into the narrow hole of a plastic insulin syringe (diameter 3.0 mm) and a copper wire was inserted from the other opening of the syringe to help connect the carbon paste with the terminal of working electrode of the potentiostat. The tip of the electrode was polished with a weighing paper until it had a shiny appearance.

The modified carbon paste electrodes were prepared by mixing graphite powder with different quantities of RGO in the same manner as bare CPE. The electrodes were packed following the same procedure described above.

### Procedure

In 10 mL volumetric flask, an appropriate aliquot of FAV working standard was inserted, and 1.1 mL of 1 mM SDS solution was added then completed to 10 mL with BR buffer and then the whole solution was transferred into the voltammetric cell. The dissolved oxygen in the solution was removed by bubbling with nitrogen for about 15 min. RGO-CPE was then immersed. The solution was stirred at 2000 rpm for the selected preconcentration period (5 s). Then, the stirrer was stopped for 5 s for solution stabilization. Cyclic and square wave voltammograms of FAV were recorded using an applied potential profile in the range from 0.5 to 1.5, 0.6 to 1.35 V respectively against 0.04 M BR buffer solution as a supporting electrolyte. The anodic peak current at RGO-CPE was measured at amplitude 0.01999 V; frequency 10.0 Hz, voltage step 0.005951 V and scan rate, 0.0595 V.s^− 1^ using SWV method against blank of the same SDS/buffer solution. Calibration curve was plotted relating the anodic peak current (*I*_*p*_) to the corresponding concentration of FAV. The regression equation and correlation coefficient were calculated.

### Spiked plasma analysis

Plasma samples were kept frozen until they were used in the test. Using a micropipette, 10 L plasma samples spiked with various aliquots of FAV were transferred into a series of 10-mL volumetric flasks. 1.1 mL of 1 mM SDS solution was added to the volumetric flask then completed to the volume with BR buffer pH 5.0. Without any further preprocessing, the solution was passed to the electrochemical cell for analysis. Then follows the procedure described as mentioned before.

### Spiked urine analysis

Until the urine samples were utilized in the test, they were stored frozen. Various aliquots of FAV in spiked 10 L urine samples were placed into 10-mL volumetric flasks. 1.1 mL of 1 mM SDS solution was transferred to the volumetric flask, and then completed to the volume with BR buffer pH 5.0. The solution was transferred directly to the electrochemical cell for examination without any extra pretreatment. Then follows the procedure described as mentioned before.

### Pharmaceutical dosage forms assay procedure

Assay for tablet: 10 tablets of FAV were accurately weighed and finely powdered. A weighed portion of this powder equivalent to 200.0 mg of FAV was dissolved in 60 mL of 0.01 M NaOH. The dissolution was enhanced via 30 min sonication then the solution was filtered. The combined filtrate and the washings (3 × 10 mL) were quantitatively transferred into a 100 mL measuring flask and complete the volume with the same solvent. The prepared solution was further diluted, then 1.1 mL of a 1 mM SDS solution was introduced to the volumetric flask, and the volume was filled with BR buffer with a pH of 5.0.

### Results and discussions

#### Surface area of electrodes

The electrochemical active surface area of RGO-CPE electrodes were evaluated using 5 mM K_3_Fe(CN)_6_ .3H_2_O as an electrochemical redox probe in 0.1 M KCl as a supporting electrolyte. The cyclic voltammogram was recorded at different scan rates and the peak current (I) was plotted against the square root of the scan rate (v^1/2^). For a reversible process, the relationship between the I and v^1/2^ is linear and controlled by Randles–Sevcik Eq. (1) [36].

1 $${\text{I}}_{\text{p}\text{a}}=(2.69 \times {10}^{5}) \times \text{A}\times {\text{n}}^{3/2}\times {\text{Do}}^{1/2}\times \text{Co}\times {{\upnu }}^{1/2}$$

Where, *I*_*p*_ refers to the anodic peak current in A, n is the number of electrons transferred, A is the electrode surface area in cm^2^, D_o_ is diffusion coefficient in cm^2^ s^− 1^, ν is the scan rate in Vs^− 1^ and C_o_ is the molar concentration of K_3_Fe(CN)_6_.3H_2_O. The diffusion coefficient of K_3_Fe(CN)_6_ in 0.1 M KCl electrolyte is 7.6 × 10^− 6^ cm^2^ s^− 1^ [37, 38]. The active surface areas of the plain and modified electrodes were calculated and found to be 0.042, 0.048, 0.051, 0.083, 0.078 cm^2^ for CPE and 2%, 5%, 7% and 10% w/w RGO modified electrodes, respectively. RGO (7%, w/w) modified electrode exhibited the highest active surface area.

### Voltammetric behavior of FAV at various electrodes

The voltammetric behavior of FAV was investigated at plain CPE and RGO-CPE in BR buffer. FAV exhibited an anodic peak at about 1.19 V, with no cathodic peak in the reverse scan, indicating irreversible electrode response (Fig. S1). The highest anodic peak current was obtained using RGO (7%, w/w)/CPE electrode, which possess the highest electrode surface area and subsequently leads to enhanced electron transfer with high electrical conductivity. Therefore, all subsequent measurements were carried out using RGO (7%, w/w)/CPE.

### Optimization of experimental conditions

#### Effect of pH

**Fig. 2:**
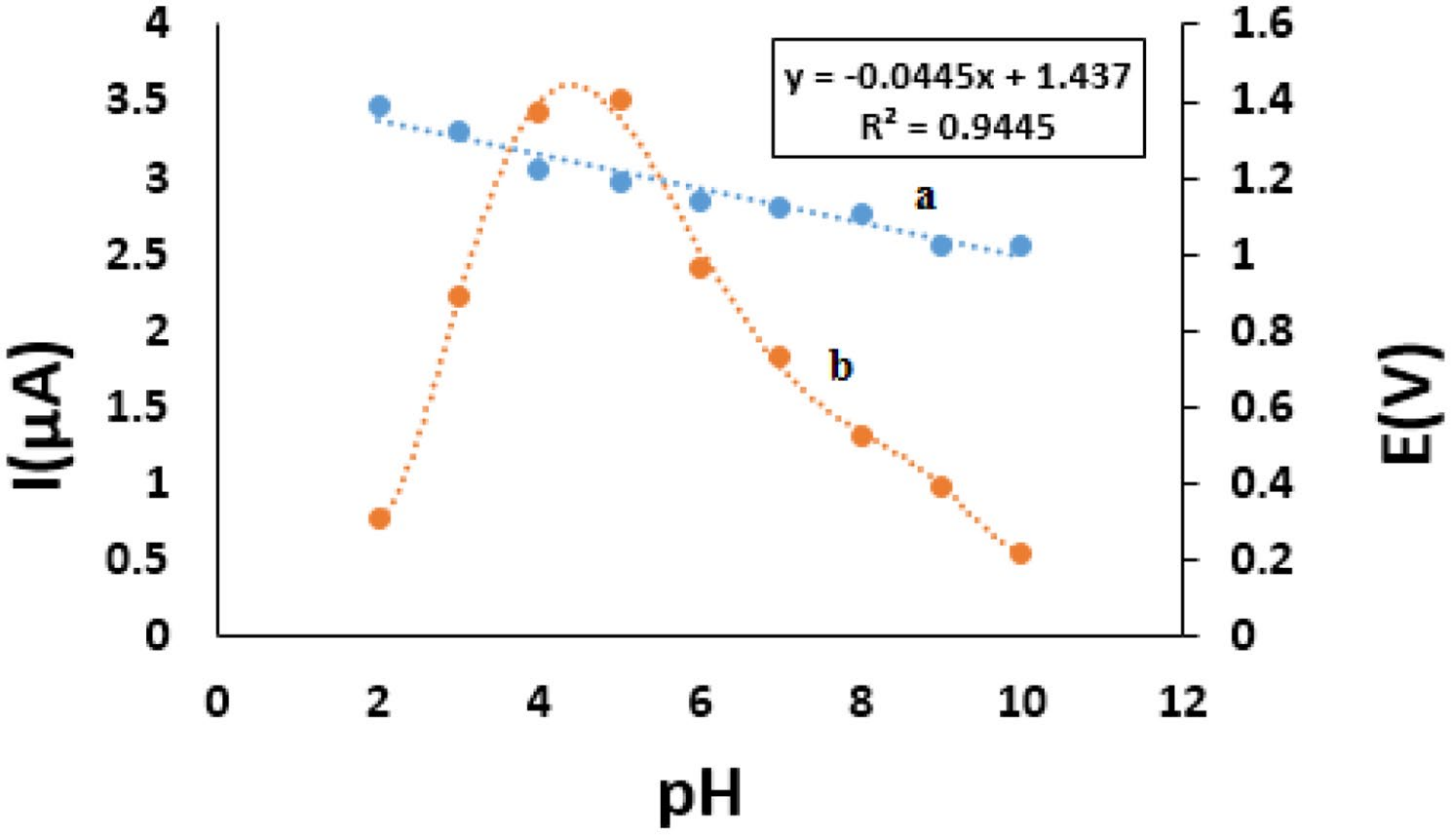
Plot of the relation between anodic peak potential E (V) and pH for of 200 ng/mL FAV at 7% RGO using SWV (a) and the effect of pH on peak current I (μA) (b)

To quantitatively reflect the rules of pH responsive electrochemical behaviors, SWV was chosen to depict the variation of the anodic peak potential in the whole pH interval since this technique has the ability of minimizing the charging current and extracting faradaic current which leads to improved sensitivity and greater accuracy. The pH of the electrolyte medium greatly affects the existing form of FAV (Keto-enol) [39] and considered as one of the variables that alters the shape of the voltammogram frequently and significantly, thus it was necessary to assess the pH impact on the voltammetric behavior of the drug. The influence of the pH on FAV anodic oxidation was investigated over the pH range 2.0–10.0. The maximum anodic peak current was recorded at pH 5, which is close to the pK_a_ (5.1) of FAV [39, 40]. The anodic peak current increased gradually with increasing the pH from pH 2.0 to 5.0 after which it decreased dramatically till pH 10.0 (Fig. 2). The anodic peak potential is shifted cathodically with increasing the pH, indicating the ease of the electron abstraction in weak acidic or neutral media. The relationship between the pH and the peak potential was found to be controlled by the following Eq. (2) in the pH range from 2.0–10.0.

2 $${\text{E}}_{\text{p}} \left(\text{V}\right)=1.437-0.0445 \text{p}\text{H} ({\text {R}}^{2}=0.9445)$$

The slope was about – 0.0445 V/pH slight lower than the Nernstian expected value for pH-effect of -0.059 V/pH unit, indicating an n-electron n-proton redox process.

### Effect of scan rate

**Fig. 3:**
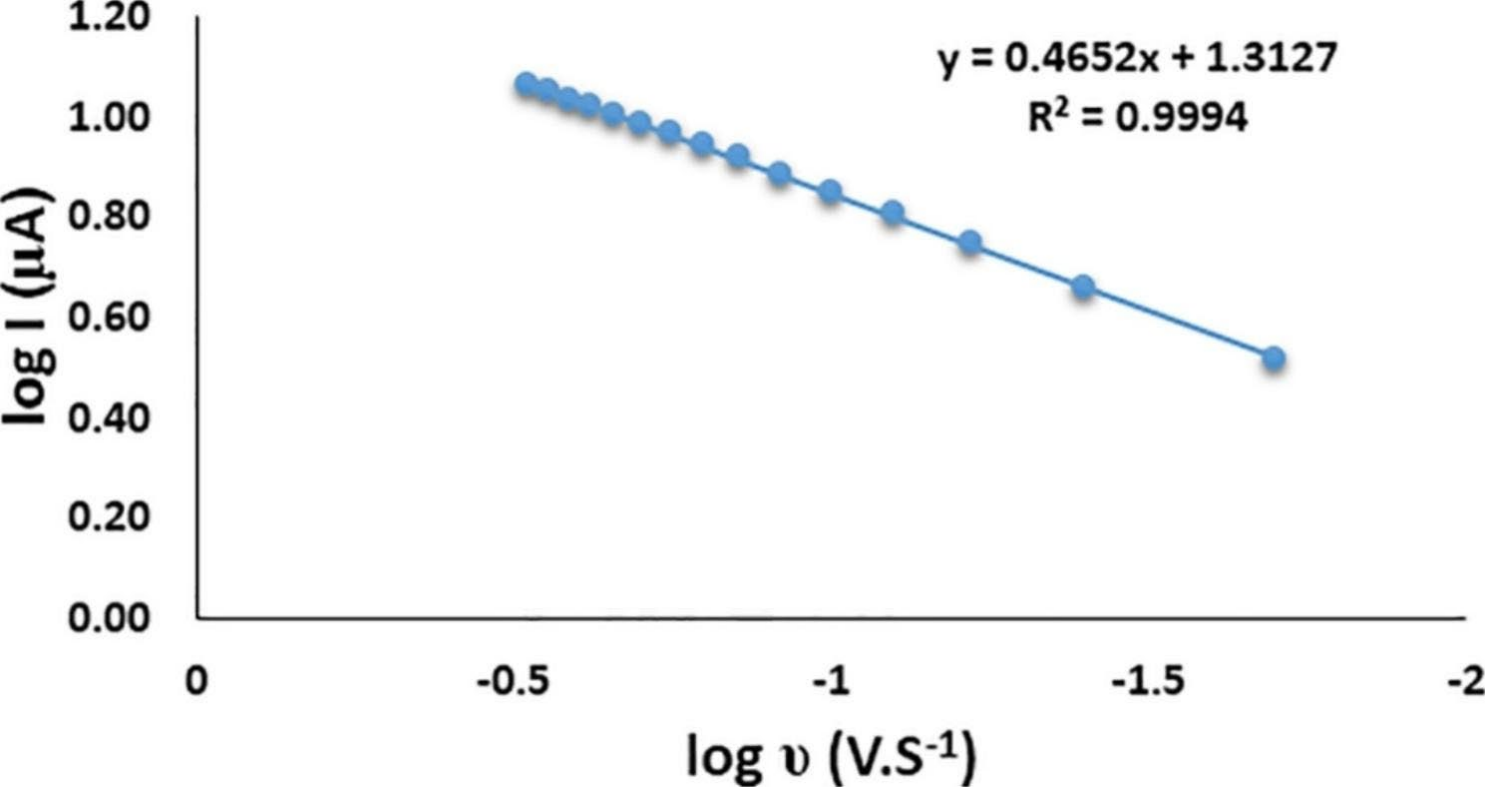
Plot of the relationship between log anodic peak current log I (µA) as a function of log scan rate log υ (v.s^− 1^) by cyclic voltammetry of 200 ng/mL FAV at 7% RGO, at pH 5.0

The relationship between the scan rate (υ) and the anodic peak current provides useful evidence, concerning the type of the process that controls the transfer of FAV to the electrode surface. The effect of υ on the anodic peak current is studied in the scan rate range from 0.02 to 0.3 V.s^− 1^. Figure 3 represents the relationship between the logarithm of the anodic peak current of FAV vs. logarithm of scan rate (log I vs. log υ). The relationship between log I vs. log υ was found to be linear and represented by the following Eq. (3):

3 $$\text{L}\text{o}\text{g} {\text{I}}_{\text{p} }\left({\upmu }\text{A}\right)=0.4562 \text{l}\text{o}\text{g}{\upupsilon } \left(\text{V}.{\text{S}}^{-1}\right)+1.3127 ({\text{R}}^{2}=0.9994)$$

The value of the slope (0.4562) is close to 0.5, indicating that the electrochemical oxidation of FAV in BR buffer is a diffusion-controlled process [41].

**Fig. 4:**
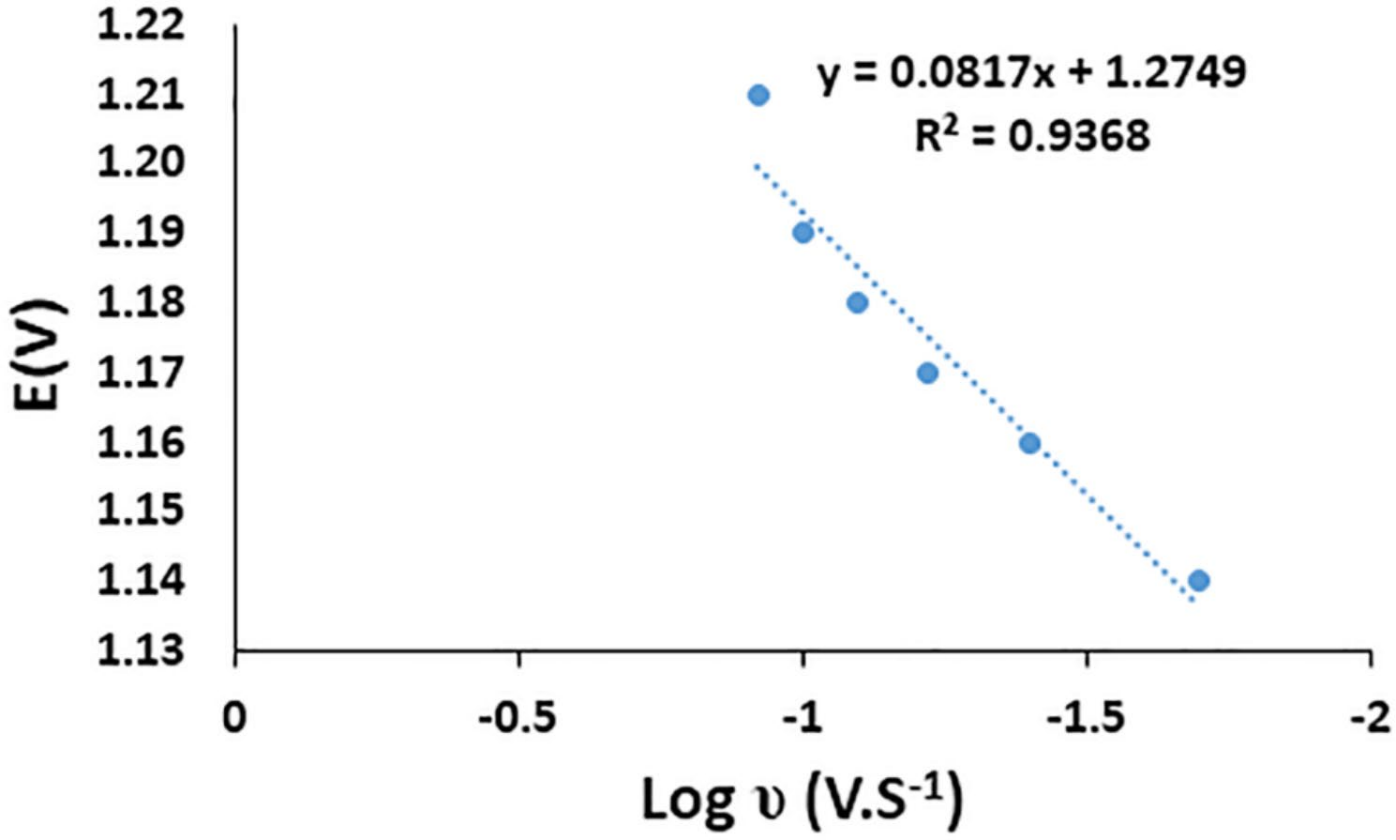
Plot of the relationship between the oxidation peak potential E (V) and. the logarithm of the scan rate log υ (v.s^− 1^)

The number of the electrons implicated in the electrochemical redox process can be estimated via Laviron equation [42] which describes the relationship between the oxidation peak potential and scan rate for the entirely irreversible electrode process, as presented in the following Eq. (4):

4 $${\text{E}}_{\text{p}}={\text{E}}^{\text{o}}+\left(\frac{2.303\text{R}\text{T}}{{\alpha }\text{n}\text{F}}\right)\text{log}\left(\frac{\text{R}\text{T}{\text{K}}^{\text{o}}}{{\alpha }\text{n}\text{F}}\right)+\left(\frac{2.303\text{R}\text{T}}{{\alpha }\text{n}\text{F}}\right)\text{l}\text{o}\text{g}\text{v}$$

where E° is the formal potential, R is the universal gas constant (8.314 J mol^− 1^ K^− 1^). T is the absolute temperature (298 K), n is the number of electrons. F is the Faraday constant (96.480 C mol^− 1^). k° is the standard heterogeneous rate constant of the reaction (s^− 1^), α is the transfer coefficient and *ν* is the scan rate (V s^− 1^). The relationship between the oxidation peak potential (*E*) versus the logarithm of the scan rate (log υ) was illustrated in Fig. 4. The resultant slope was 0.0817 and (αn) was calculated to be 0.722, α is considered to be 0.5 for irreversible anodic peak. Therefore, the value of n was found to be = 1.44 (n ≈ 1), thus the electrochemical (one-electron) oxidation mechanism would most likely follow Scheme S1.

### Effect of SDS

The effect of SDS as an anionic surfactant on the electrochemical oxidation of FAV in BR buffer pH 5.0 was investigated using SWV. The SDS critical micelle concentration was reported to be 8.5 mM [43] though a lower SDS concentration (1 mM) was added in various volumes to the electrochemical cell containing FAV. It was observed that 1.1 mL was the optimal volume for achieving the highest anodic peak current for FAV (Fig. S2). The reason for this is the formation of a complex between the drug molecules and the monomers of SDS surfactant so SDS diffuses into the hydrophobic electrode along with FAV and consequently its electrode surface concentration greatly improves which in turn results in increasing the signal [44, 45]. So, SDS should be in the monomer form to be available for complexation with FAV molecules. This hypothesis was supported by simulations of molecular dynamics and interactions using molecular modeling software®, which confirmed the formation of a complex between FAV and SDS, as shown in Fig. S3 (a) and (b).

### Validation of the proposed method

The proposed SWV method was validated according to the International Conference on Harmonization (ICH) criteria [46]. Validation parameters include linearity, range, LOD, LOQ, precision, accuracy, robustness, and specificity.

### Linearity and range

**Fig. 5:**
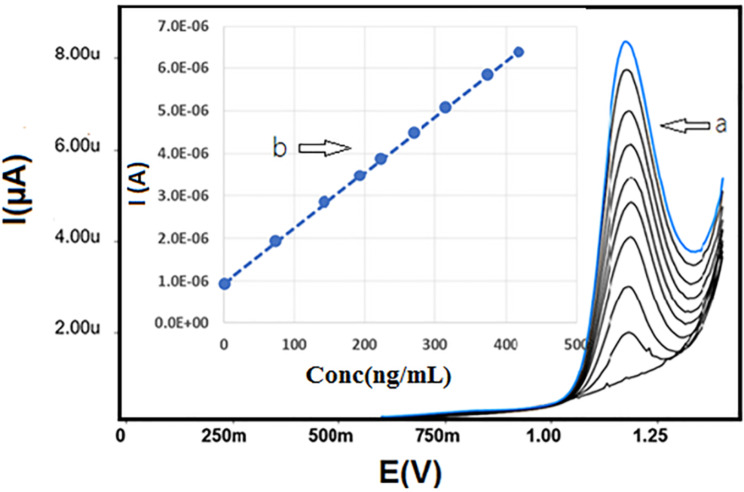
SWV voltammograms of FAV at the RGO in 0.04 M BR buffer solution pH 5 (with SDS) (**a**) and, plots of linearity of various concentrations of FAV concentrations and the corresponding current (**b**)

The SWV method was found to be linear in the concentration range of 1.5–420 ng/mL (Fig. 5). The linearity parameters in different matrices are summarized in Table 1.

**Table 1:** Analytical parameters for the determination of FAV at RGO/CPE in pure form, spiked human plasma and urine by using SWV method

Parameters	Pure form	Plasma	Urine
Linear range (ng/mL)	1.5–420	20–350	25–350
Slope (ng/mL)	0.013	0.011	0.012
Intercept	0.935	0.986	0.945
Correlation coefficient (r)	0.9998	0.9998	0.9997
LOD (ng/mL)	0.44	5.13	7.96
LOQ (ng/mL)	1.34	15.57	24.13

### Limit of detection (LOD) and limit of quantitation (LOQ)

The lowest amount of analytes that can be identified but not necessarily quantified as accurate quantities was used to determine the LOD. The lowest amount of analytes that can be quantitatively identified with sufficient precision and accuracy was used to estimate the LOQ. Table 1 shows a summary of the findings. According to ICH guidelines, the LOD and LOQ were determined using the following equations:

$${\rm{LOD = 3.3 SD/S}}$$

$${\rm{LOQ = 10 SD/S}}$$

Where SD is the standard deviation of the intercept and S is the slope of the linear calibration curve.

### Accuracy

The accuracy was assessed at five concentration levels covering the specified linear range in triplicate. The average recovery percentage was 99.36% (Table S1), indicating a satisfactory accuracy of the results.

### Precision (repeatability and intermediate precision)

The intra- and inter-day precision were assessed on the same day and on three different days, respectively. The analysis was carried out using triplicate preparations of FAV at three different concentration levels (20, 100, 300 ng/mL) within FAV range. The results (Recovery ± SD, RSD) show that the proposed method is precise (Table S2).

### Robustness

It was investigated if an analytical procedure can stay unaffected by minor changes in experimental conditions. The stability of the anodic peak current was tested with slight variations in experimental parameters such as electrolyte pH 5 ± 0.2 in order to verify the method robustness. The average recovery was not less than 99% and not more than 102% with %RSD < 1, indicating a satisfactory robustness of the proposed SWV method (Table S3).

### Specificity

The ability of the described voltammetric technique to quantify FAV in a pharmaceutical formulation without interference from typically present excipients and additives demonstrates its specificity. The influence of common tablet excipients and electroactive biological compounds were investigated. The tested excipients included mannitol, magnesium stearate and carboxy methyl cellulose sodium. Results showed that the variation in the SWV peak height for FAV was less than 2% in presence of these compounds. The experimental interference results are summarized in (Fig S4). Additionally, the potential electroactive biological interferants as ascorbic and uric acids were investigated. These compounds were used in concentrations higher than that of FAV. It was observed that the peak of FAV was well separated with a little increase (< 2.5%) in the peak height of FAV, (Fig S4, S5). The method was also effectively applied for evaluation of cited drugs in spiked human urine and plasma with high recovery and low matrix effect as displayed in Table 2.

**Table 2:** Application of the proposed method for the determination of FAV in dosage form, plasma, and urine

Parameters	Dosage form		Plasma		Urine	
Amount taken (ng/mL)	Amount found (ng/mL)	Recovery (%)	Amount found (ng/mL)	Recovery (%)	Amount found (ng/mL)	Recovery (%)
20	19.93	99.69	19.80	99.00	19.70	98.50
80	80.68	100.86	78.54	98.18	78.46	98.08
150	148.43	98.95	149.46	99.64	151.50	101.00
250	251.26	100.50	251.45	100.58	251.22	100.49
350	349.25	99.78	350.76	100.22	350.78	100.22
Mean ± S.D		99.96 ± 0.75		99.52 ± 0.96		99.66 ± 1.29

### Applications

#### Determination of FAV in Avipiravir® tablets

The ability of the methods to quantify FAV in its commercially available pharmaceutical formulations (Avipiravir® tablets) was tested, and no interference from excipients was found. The recovery percentages (Table 2) are satisfactory. The developed methods were also shown to be highly accurate in recovery studies.

#### Determination of FAV in spiked urine and plasma

The suggested method after sample pretreatment as discussed above was successfully used to quantify FAV in spiked human urine and plasma. Table 2 displayed the recovery percentages of FAV in the spiked human plasma and urine. The obtained results revealed no significant matrix effect.

### Comparison to reported method

The validated methodology suggested were used to determine the drug in its marketed tablet dosage form (Avipiravir tablet), and the findings were statistically compared to the published HPLC technique [14]. As shown in Table 3, a statistical comparison of the results obtained using the recommended strategy, those acquired using the reported method using Student’s t test, and variance ratio F-test revealed no significant difference between the two techniques. To evaluate the data visualization, some statistical tools were used (Fig. S6). Interval plots (Fig. S6, a) depict data as an interval, with the central point representing the interval mean. The intersection of the proposed method interval and the reported one confirms the t-test and F-test results, indicating that there is no significant difference between the two groups. Another interesting tool for data visualization is the Boxplot (Fig. S6, b), which depicts the distribution of data between groups. The central quartile is represented by the central box, which has a line representing the data median, upper lines that represent higher values, and whiskers that represent lower values. The boxplot depicts the distribution of data in each data group. Another tool for determining if data is normally distributed is the normal probability plot. The normal distribution in the data (Fig. S6, c) is satisfied as the straight line passes through the majority of the data points. Interval plot, boxplot, normal probability were achieved using Minitab software.

**Table 3:** Statistical analysis of the results obtained by the proposed (SWV) method for determination of FAV in dosage form at RGO/CPE

Parameters	Avipiravir tablet®	
	Proposed method	Reference method^c^ [14]
Mean^a^ ± S.D.	99.96 ± 0.75	99.25 ± 1.51
*t*-test	0.95 (2.36)^b^	
*F*-test	4.05 (6.39)^b^	

^a^ Mean of five determinations

^b^The values in parenthesis are the corresponding theoretical values of t and F at P = 0.05

^c^ Waters Alliance 2695 high-performance liquid chromatography (HPLC) system equipped with a quaternary gradient pump, autosampler and photodiode array detector 2996, Waters VDSpher PUR 100 C18-E column (5 μm, 250 × 4.6 mm)

## Performance in terms of greenness

**Fig. 6:**
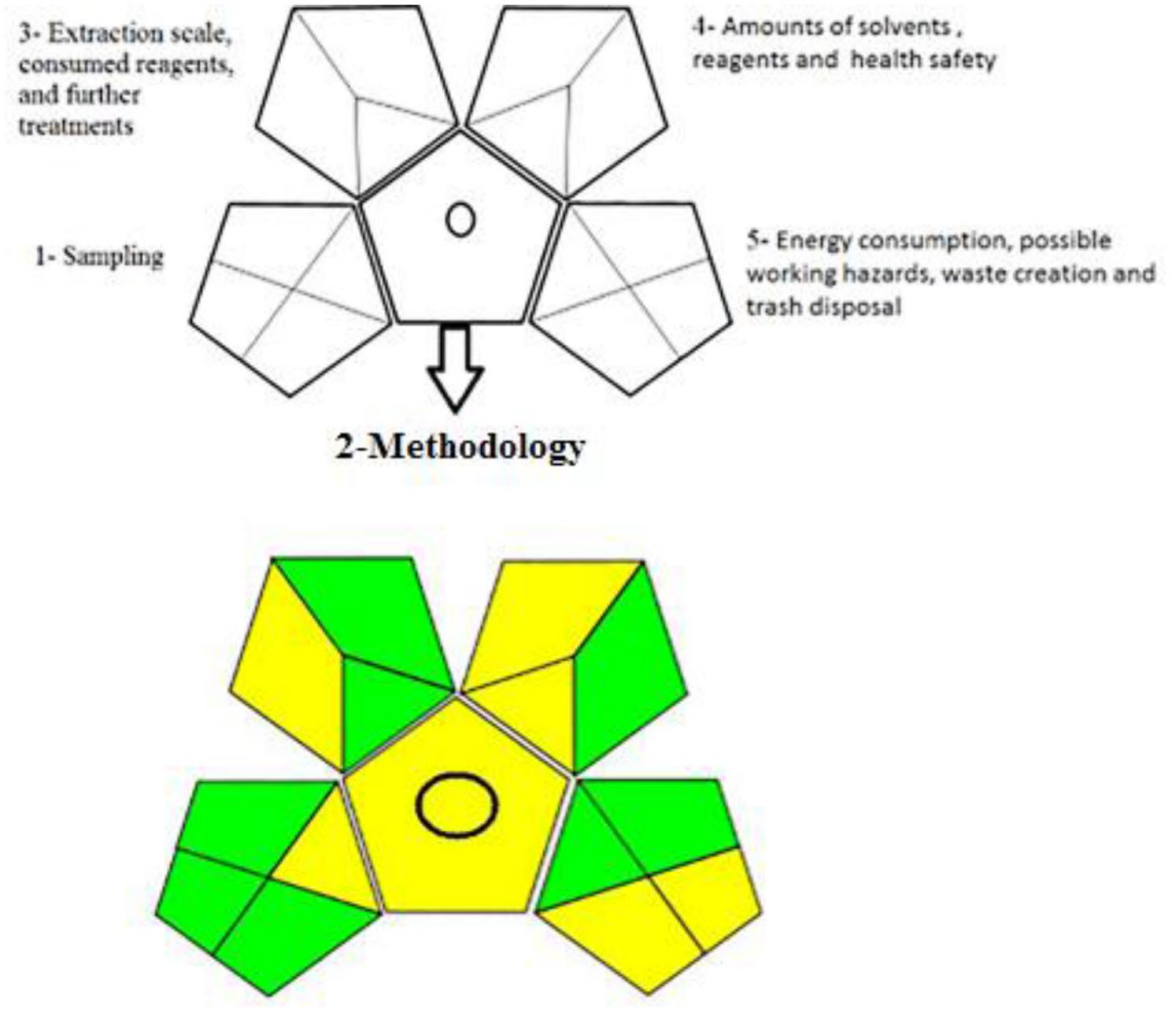
Greenness evaluation of proposed method using GAPI index

The environmental performance of any established analytical method nowadays must be taken in consideration. This new tendency piqued our interest, and we realized its significance in our research. As a result, we conducted a greenness assessment of the proposed (SWV) technique in order to determine the method hazardous impact on the analyst and the environment, as well as to identify the weakest spots that may be improved in order to meet the green analytical chemistry requirements. GAPI is a new and frequently used measure for evaluating analytical procedures. The GAPI pictogram is made up of 15 components grouped into five primary pentagrams, each representing an analytical step. GAPI assesses ecological effect using three colors (green, yellow, and red), with red indicating bad effect, yellow indicating a moderate effect, and green indicating a low environmental effect. This first, four-fielded pentagram represents sampling. The second pentagram, which has just one field, represents the methodology type. The third pentagram is comprised of three fields: extraction scale, consumed reagents, and further treatments. The fourth pentagram addresses the amounts of solvents and reagents used as well as the hazards to one’s health and safety. The fifth pentagram addresses the tool’s energy consumption, possible working hazards, waste creation and trash disposal [30, 47–49]. Figure 6 shows 8 green 7 yellow, and no red pentagrams in the proposed method.

## Conclusions

In the present study, an ecofriendly method and a sensitive electrochemical sensor was developed for FAV determination in pharmaceutical and biological fluids. A surfactant-containing solution improve the sensitivity of the electrochemical sensor composed of CPE modified with RGO nanoparticles. The molecular dynamics simulations support that FAV-SDS interaction. A decrease in the potential along with concomitant increase in the anodic peak current can be achieved with 7% RGO modified electrode. The method displayed a high sensitivity with a detection limit of 0.44 ng/mL. The developed method offers an inexpensive, simple, and rapid analysis where no sample pretreatment was needed. Furthermore, the method demonstrated satisfactory accuracy and precision for determination of FAV. FAV determination was not affected by the presence of two potential electroactive interfering substances, the uric acid which may increase during FAV therapy and the recommended co-administered vitamin C. The method complies with GAPI metrics regarding the safety to analyst and environment.

### Electronic supplementary material

Below is the link to the electronic supplementary material.

Supplementary Material 1

## Data Availability

All data generated or analyzed during this study are included in this published article.
