# Supplementary material for: A highly sensitive and green electroanalytical method for the determination of favipiravir in pharmaceutical and biological fluids
Source: BMC Chem. 2023 Aug 31;17(1):109. doi: 10.1186/s13065-023-01023-z (PMC10472665; doi:10.1186/s13065-023-01023-z)
Supplement: Supplementary file 1 — Supplementary Material 1 [file 13065_2023_1023_MOESM1_ESM.docx]

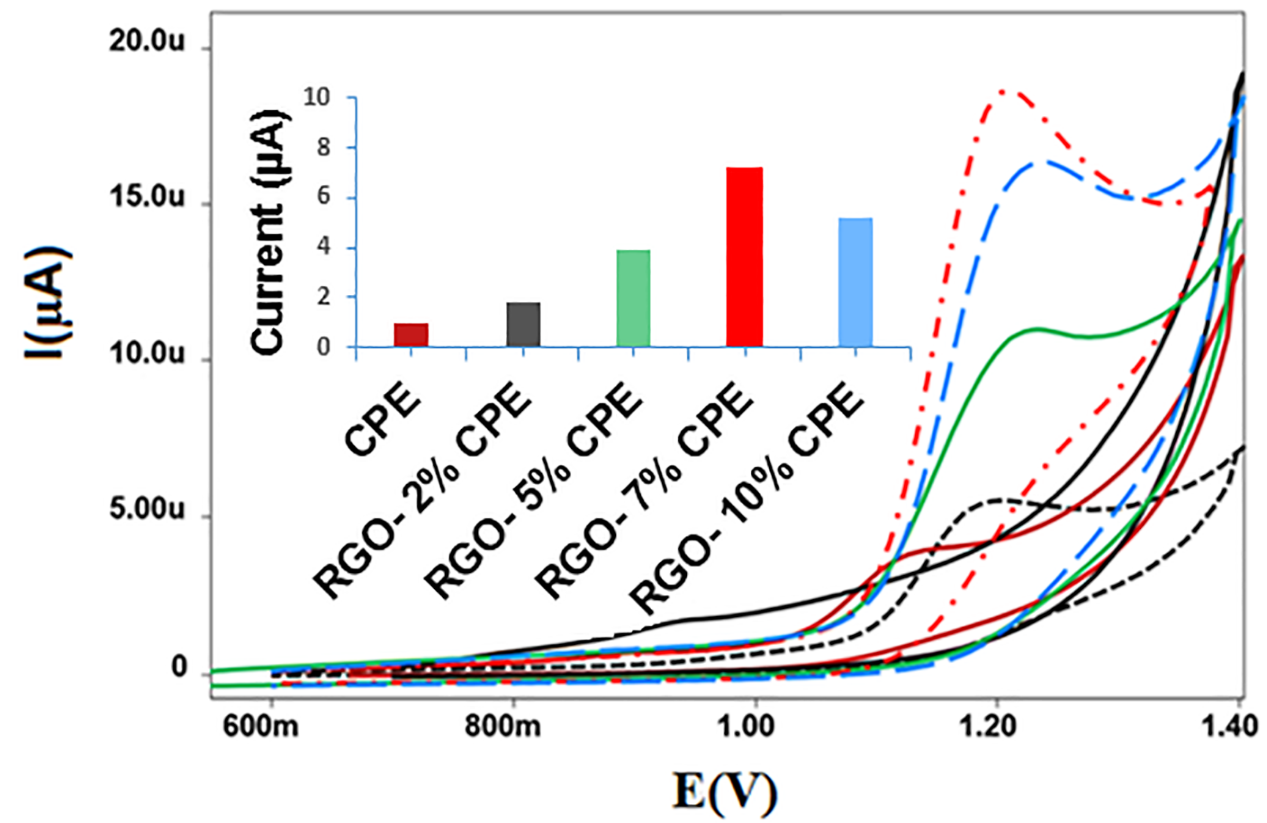


Fig. S1: Comparison of CV voltammograms response at different electrodes of 200 ng/ml FAV at pH 5 at 100 mV/S


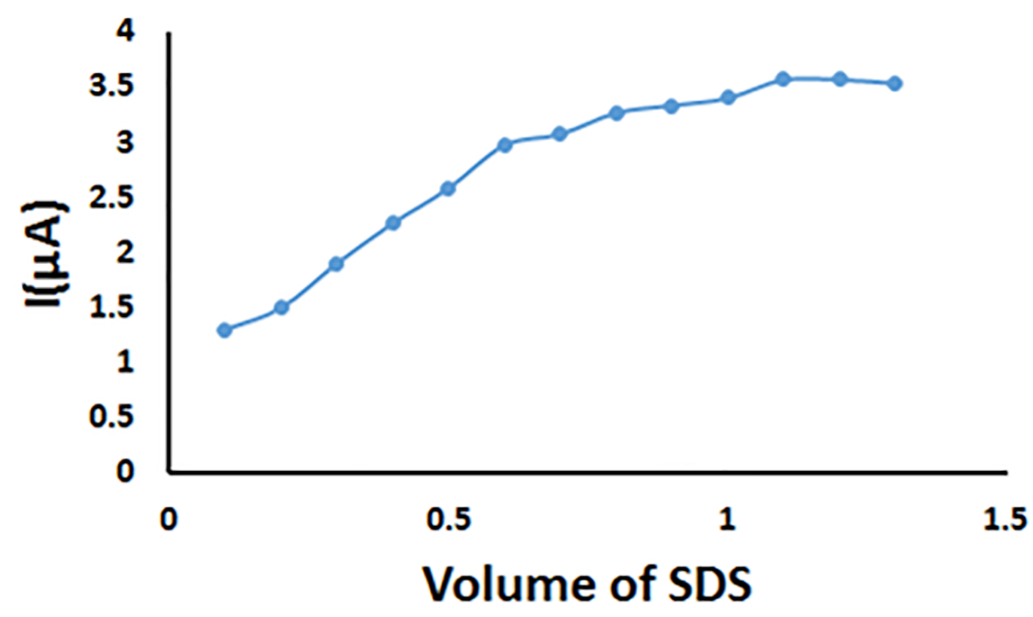


Fig. S2: The effect of volume of SDS surfactants on peak current of FAV solution (SDS-1 mM) in 0.04 M BR buffer


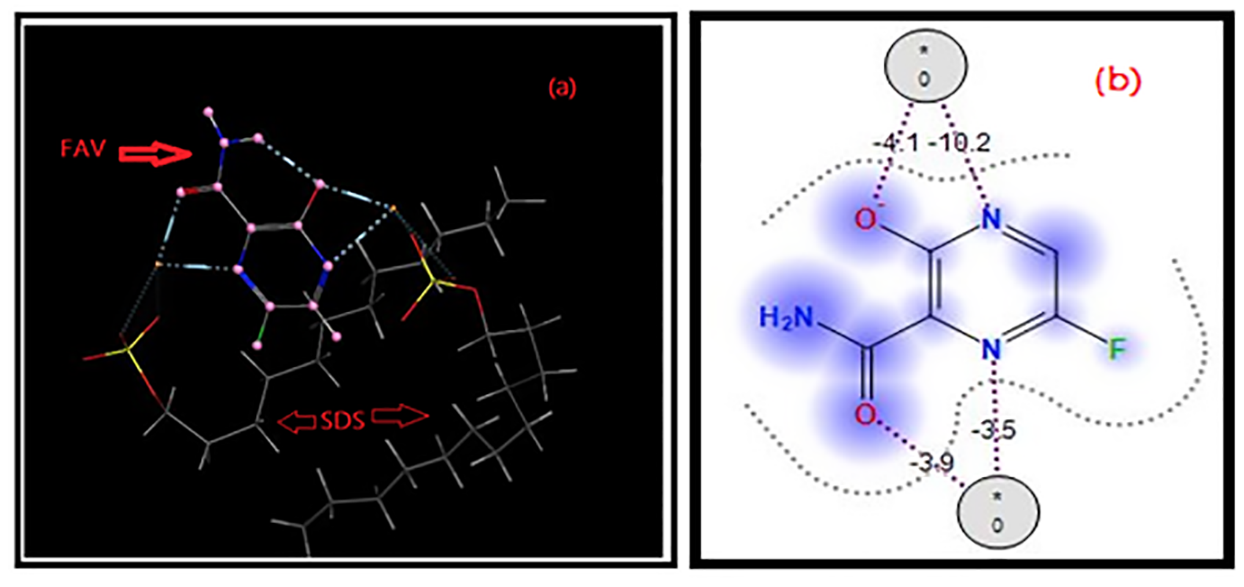


Fig.S3: FAV in the vicinity of SDS molecules (a), and molecular interactions between FAV and SDS (b)


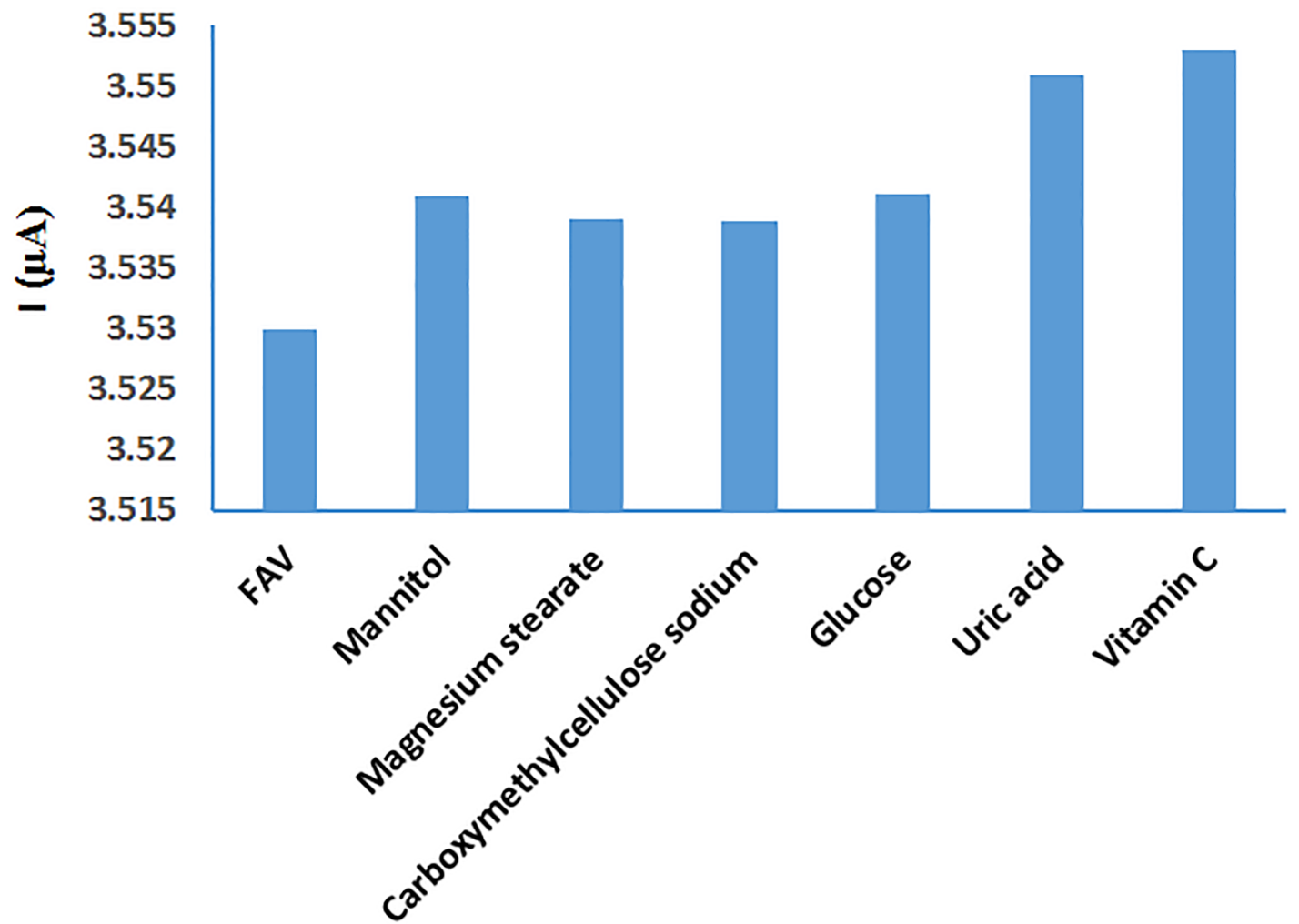


Figure S4. Effect of different interfering materials (5 x 10^-5^ M) on FAV (200 ng/mL) in a pH 5.0 B.R. buffer solution.


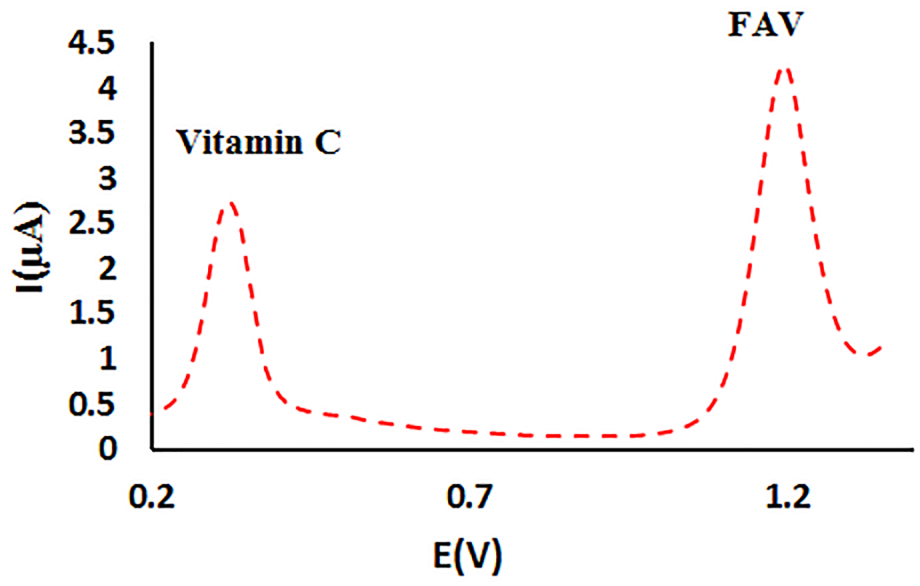

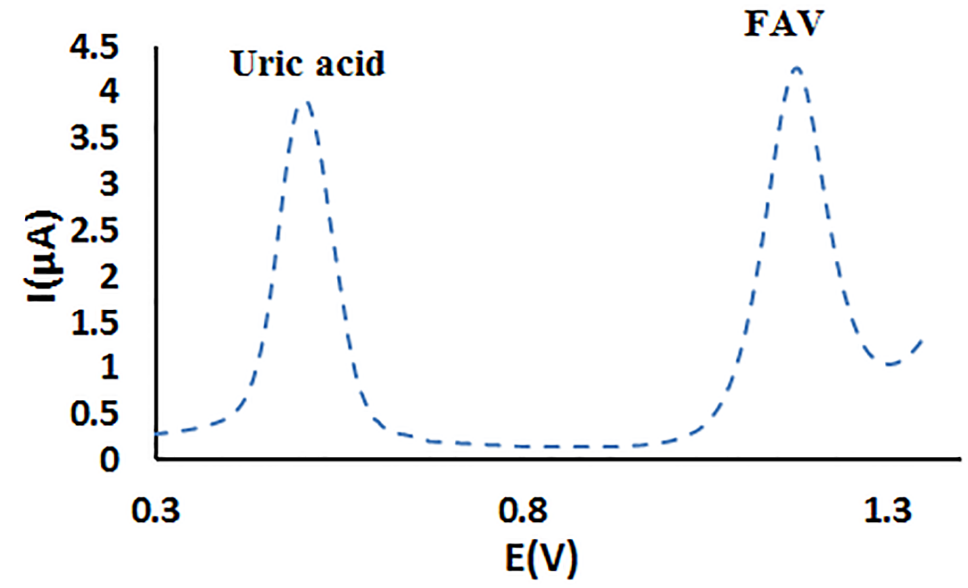


Fig. S5. SWV using RGO in a pH 5.0 B-R buffer containing 200 ng/mL FAV, 5 x 10^-5^ M for vitamin C and uric acid.


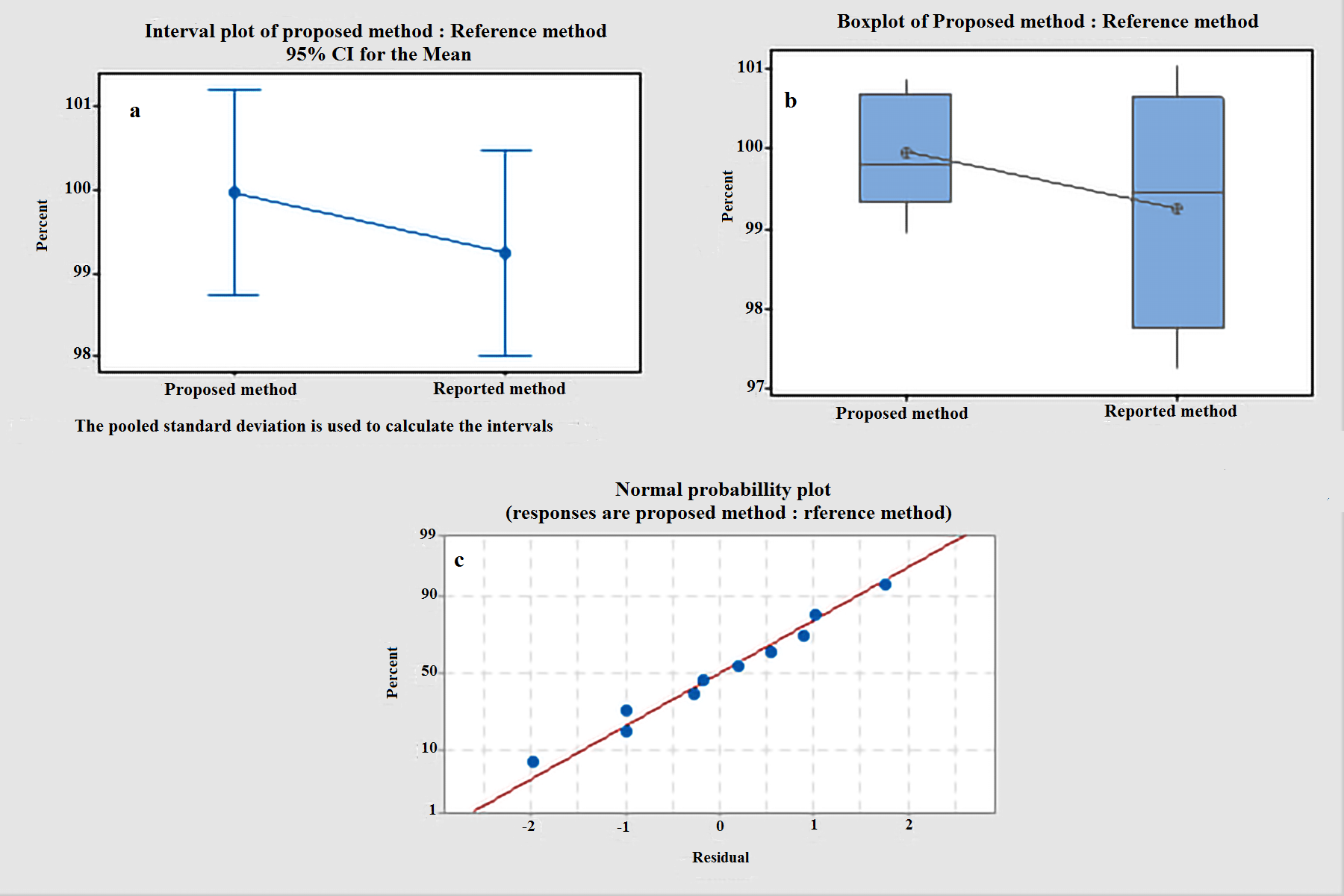


Fig. S6: Interval plot (a), Box plot (b), and normal probability plot (c) of the proposed and reported methods


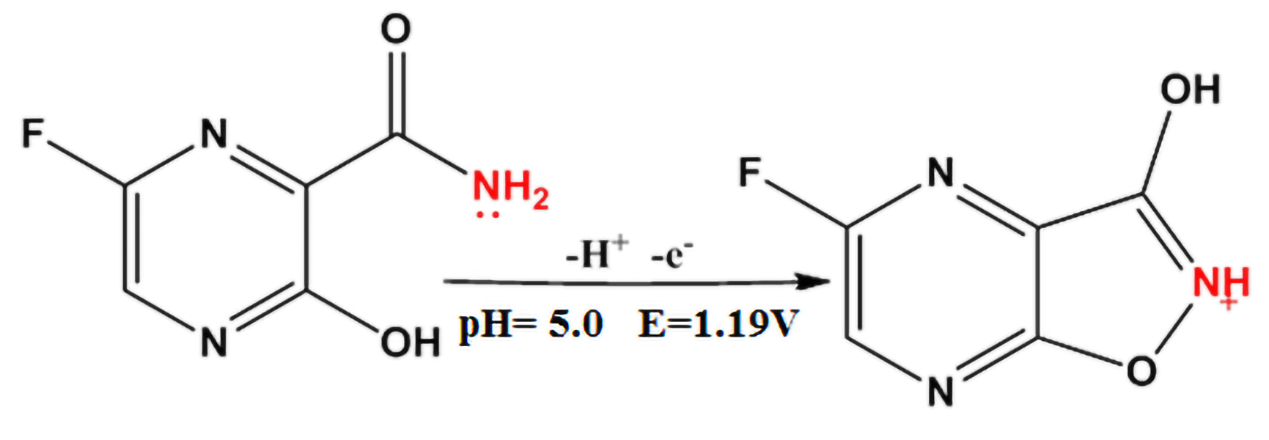


Scheme S1: proposal for the oxidation mechanism of FAV

Table S1. Accuracy results for determination of FAV in pure form using the proposed method.

| Drug | Conc.taken (ng/mL) | Conc found (ng/mL) | % Recovery^a^ | Mean ± SD | RSD % |
| --- | --- | --- | --- | --- | --- |
| FAV | 20 | 19.86 | 99.31 | 99.36 ± 0.69 | 0.69 |
|  | 80 | 80.07 | 100.09 |  |  |
|  | 150 | 149.24 | 99.49 |  |  |
|  | 250 | 249.24 | 99.69 |  |  |
|  | 350 | 343.87 | 98.24 |  |  |

^a^ Mean of three repetition

Table S2. Precision results for determination of FAV in pure form using the proposed method.

| Drug | Repeatability | | | | | Intermediate precision | | | | |
| --- | --- | --- | --- | --- | --- | --- | --- | --- | --- | --- |
| FAV | Conc. taken (ng/mL) | Found  (% Recovery) | Mean^a^ | Mean ± SD | RSD % | Conc. taken (ng/mL) | Found  (% Recovery) | Mean^a^ | Mean ± SD | RSD % |
|  | 20 | 100.88 | 100.12 | 99.75 ± 0.32 | 0.32 | 20 | 98.19 | 98.68 | 99.54 ± 0.91 | 0.91 |
|  |  | 101.19 |  |  |  |  | 99.38 |  |  |  |
|  |  | 98.31 |  |  |  |  | 98.46 |  |  |  |
|  | 100 | 98.46 | 99.51 |  |  | 100 | 100.95 | 100.49 |  |  |
|  |  | 100.76 |  |  |  |  | 100.76 |  |  |  |
|  |  | 99.32 |  |  |  |  | 99.77 |  |  |  |
|  | 300 | 99.48 | 99.62 |  |  | 300 | 98.92 | 99.45 |  |  |
|  |  | 99.54 |  |  |  |  | 99.41 |  |  |  |
|  |  | 99.86 |  |  |  |  | 100.02 |  |  |  |

^a^ Mean of three repetition

Table S3 : The suggested FAV method's robustness

| Parameters | % Recovery^a^ | % RSD |
| --- | --- | --- |
| pH 4.8 | 99.82% | 0.96 |
| pH 5 | 99.39 % | 0.68 |
| pH 5.2 | 100.57 % | 0.58 |

^a^Mean of three repetition.
